# When Collaborating in Constructive Task With Spouse: Attachment Orientation Predicts Exploratory Behavior Among Older Couples

**DOI:** 10.3389/fpsyg.2021.628412

**Published:** 2021-06-16

**Authors:** Yan Wang, Xiancai Cao, Fengzhan Li, Dahua Wang

**Affiliations:** ^1^Faculty of Psychology, Beijing Normal University, Beijing, China; ^2^Key Research Base of Humanities and Social Sciences of the Ministry of Education, Academy of Psychology and Behavior, Tianjin Normal University, Tianjin, China; ^3^Faculty of Psychology, Tianjin Normal University, Tianjin, China; ^4^Tianjin Social Science Laboratory of Students’ Mental Development and Learning, Tianjin, China; ^5^Department of Military Medical Psychology, Fourth Military Medical University, Xi’an, China

**Keywords:** attachment, exploration, actor-partner independence model, avoidance, older adults

## Abstract

In order to examine the function of the attachment system in later life, this study investigated the relationships between attachment orientation and exploratory behavior in a collaborative constructive activity with one’s spouse among older adults. In total, 49 older couples completed a collaborative constructive task, and their behaviors were videotaped. Each participant’s exploratory behavior (i.e., engagement, enjoyment, and distress) was coded by independent raters. The results revealed older adults highly engaged in the collaborative activity. In addition, based on the actor-partner interdependence model, the results indicated that attachment avoidance positively predicted the individuals’ enjoyment as well as their partners’ distress during the collaborative activity. The current findings extend the literature regarding the effects of attachment on exploration from early adulthood to later life, from the workplace to family life, and from individual’s own behavior to collaborative situation.

## Introduction

Although emotional goal is the dominant motivation in later life (Carstensen and Turk-Charles, 1994), dealing with unfamiliar environments and exploring methods to solve novel problems or manage new tasks is also an important part of daily life for older people. Various exploratory activities are undertaken by the way of involving both sides of couples as collaborators, as the spouse is the most significant others (Pinquart and Sörensen, 2011) and may play as the most frequent cooperators for older couples. Sometimes, husbands and wives need to discover new, creative, or cognitive-demanding things together (Aron et al., 2000), such as exploring a new function of a smartphone, taking part in some creative activities in community, or planning a special travel to new or old places. However, it is unclear so far to what extent they enjoy exploring activities with their partners, as well as whether attachment orientations play a role in accounting the individual differences in the exploring experiences. Our goal in this study is to fill the gap from an actor-partner perspective, using a dynamic constructive task.

According to attachment theory (Bowlby, 1969, 1982), individuals come into the world equipped with exploration, or an exploratory behavioral system, a system aimed at investigating, manipulating, and mastering the environment. Optimal functioning of the exploration system benefits us in many ways, including facilitating behavioral self-regulation, sustaining adaptation and growth, and increasing a person’s sense of competence (Mikulincer and Shaver, 2016). Attachment theory also points out that attachment figures play an important role in individuals’ lives by functioning as not only a safe haven that protects them from threats and distresses but also a secure base that is available and responsive when needed to support them to explore the external world, in the way of working, playing, discovering, goal pursuing, and taking part in activities with peers (Bowlby, 1980; Waters and Waters, 2006). Thus, it is worthy to understand how attachment affects exploration system function in our daily life.

Ainsworth et al. (1978) conducted the first study that demonstrated differences in exploration according to attachment security in infants. In line with the hypotheses of attachment theory, secure infants who viewed their mothers’ availability and responsiveness positively engaged in exploration activities with ease. In contrast, insecurely attached infants with a negative view of self or others exhibited poorly balanced dependence and exploration tendencies.

Only a few studies have investigated the relationship between attachment and exploration in adulthood, but most of them replicated the patterns that were found in childhood, demonstrating that the exploration is strongly predicted by individuals’ attachment orientation which links with the extent they percept their partner as secure base (Feeney and Thrush, 2010; Jakubiak and Feeney, 2016). For example, several cross-sectional studies have shown that attachment insecurity led to poor performance (Neustadt et al., 2011) and maladjustments in the workplace (Hazan and Shaver, 1987; Schirmer and Lopez, 2001; Pines, 2004; Leiter et al., 2015), which can be regarded as a typical exploration in adulthood.

According to attachment theory (Bowlby, 1969, 1973; Ainsworth et al., 1978), an individual’s exploratory behavior is highly depended on the secure base function of the attachment figure, and secure partners are more likely to provide secure-based supports, including availability, encouraging goals, and not interfering (Feeney and Thrush, 2010). However, most of these studies assigned one partner of the couple as an explorer and only focused on the intrapersonal effect of attachment (i.e., how individual’s attachment influences his/her own exploration) without the consideration of the effects of relationship partner (e.g., Kanat-Maymon et al., 2012; Leiter et al., 2015; Jakubiak and Feeney, 2016). Recently, one study focused on younger couples has examined the partner effect of attachment on exploration (i.e., Coy et al., 2012). Consistent with the secure base function of attachment system, this study showed that an insecure (especially anxious) partner was more likely to hinder individual’s exploration. Specifically, individuals with a highly anxious partner, who were less available and more interfering, could feel less positive after doing mediation (regarded as an exploration activity in the study) in the presence of their partner than doing that alone (Coy et al., 2012).

### Attachment and Exploration in Later Life

To the best of our knowledge, most research (either focused on actor effect or partner effect of attachment) has investigated the function of attachment on exploration among younger population (e.g., children and young adults). To date, only one study focused on older adults (Jakubiak and Feeney, 2016). Consistent with attachment theory (Bowlby, 1980), this study revealed that older adults with greater avoidance reported less daily goal progress. However, the exploratory behavior was assessed by only one self-reported item which might be influenced much by consciousness and self-regulation. Furthermore, this study only revealed the actor effect of attachment on exploration. There is still little known about whether and how older adults’ attachment orientations influence the way they themselves and their partner behave during exploration. Moreover, previous findings from childhood and early adulthood may not be applied to older adults as attachment research has revealed some aging effects.

First, the typical and common exploratory activities among older people may differ from those of their younger counterparts. Although the socioemotional selectivity theory (Carstensen, 1987, 1992) points out the older adults’ motivation to obtain novel information and acquire self-identity decreases, it does not mean that older adults no longer engage in exploratory activities. Instead, most of the novelty seeking remained stable or increased as aging (Reio and Choi, 2004), which arouses environmental exploration (Cahill-Solis and Witryol, 1994). Moreover, according to Bowlby, 1969, 1982, when people encounter novel or unexpected stimuli or conditions that challenge their knowledge, beliefs, or actions, the exploratory behavioral system will be activated and allow individuals to investigate, manipulate, and master the environment. Thus, though older adults no longer participate in school, work, and alike exploration that is typical in younger age, they still need to manage novel or cognitive-demanding tasks or to solve problems in daily life (Fitzpatrick, 2009). For example, crafts, dancing, and using Web-based devices become common exploratory activities in older adults.

In addition, to achieve and maintain positive affect, older adults usually reduce their indifferent social network and spend more time with their significant others, such as spouse (Fredrickson and Carstensen, 1990; Lang and Carstensen, 1994; Antonucci et al., 2004; Fung and Carstensen, 2006). Thus, most of the exploratory activities are undertaken by the way of involving both sides of the couple as collaborators (Fitzpatrick, 2009). However, in the exploratory context induced in most of the previous research, one partner of the couple was usually assigned as the explorer and the interaction within the couple was not allowed (e.g., Feeney and Thrush, 2010; Coy et al., 2012). The findings about such non-interactive exploration may not be applicable to understand older adults’ exploratory behavior while collaborating with their partners. Furthermore, the extant study on older adults (e.g., Jakubiak and Feeney, 2016) assessed only the actor effect of attachment, yet one’s exploratory behavior might be greatly affected by their partner during this kind of joint activity (Bowlby, 1980). Therefore, research considering collaboration as well as actor and partner effects is required to reveal the relationship between attachment and exploration in later life.

Second, regarding attachment, older adults tend to exhibit a different pattern from their younger counterparts (van Assche et al., 2013). In later life, older people who experience declines in resources tend to defensively place more emphasis on independence and self-reliance and less emphasis on interdependence; thus, they generally become more avoidant and less anxious (Cusimano and Riggs, 2013; Chopik and Edelstein, 2014). Additionally, previous research has found that, among older adults, attachment avoidance (not anxiety) is influential for individual’s behavior, such as perception of social support (Kafetsios and Sideridis, 2006) and relationship-related information processing (Wang et al., 2017). Accordingly, the level of avoidance may become salient when predicting exploratory behavior among older adults. Specifically, we hypothesized that compared to anxiety, attachment avoidance may have stronger negative effects on both individuals’ and their partners’ exploration.

### Current Study

The current study was based on attachment theory and aimed to examine the associations between attachment orientations and exploratory behaviors in a collaborative activity in married older adults, from a dyadic perspective.

A cooperative sand-play task was designed for this study to observe exploratory behaviors within an older couple. During this task, older couples needed to jointly construct a creative scene with a variety of miniature figures/objects on the sand. They were asked to take action (e.g., place a new miniature figure into the sand tray or move a miniature object already in the sand tray) in turns. This task was used in this study because (1) it was considered as a typical collaborative and exploratory situation for older adults as it is novel for the participants and the couple needs to construct, discover, and pursue goals together (Bowlby, 1980); (2) both couple members acted as explorers who had to take actions during the activity, which made it possible to code and analyze both partners’ exploratory behaviors; and (3) since the interactions between the two couple members were allowed (though it was not explicitly requested), it was a better task to mimic the collaborative activities in real life than the tasks employed in previous studies (e.g., Coy et al., 2012). Regarding the exploratory behavior, we employed a comprehensive coding system and assessed eight types of specific behaviors that would extend the previous findings on self-reported outcome of exploration in older adults (Jakubiak and Feeney, 2016).

What is more, much of what is known about attachment orientations and exploration among older adults has been studied at the individual level (e.g., Jakubiak and Feeney, 2016); however, individuals’ attachment orientations can be highly likely to influence not only their own but also their partners’ behaviors (Kane et al., 2007; Sadikaj et al., 2017). The actor-partner interdependence model (APIM; Kenny et al., 2006) was utilized to examine the potential effects of attachment on individuals’ behaviors (i.e., the actor effect) and that of their partners (i.e., the partner effect). According to the attachment theory (Bowlby, 1980), secure individuals tend to interact well with their partners (e.g., providing enough and suitable supports) and function well as a secure base (e.g., Kane et al., 2007), which in turn can promote their partners’ engagement in exploration with a feeling of security. In contrast, insecure individuals are likely to be somewhat insensitive to their partners’ needs (Feeney and Thrush, 2010) and do not manage well during disagreements or conflicts with their partners (Beck et al., 2013; González-Ortega et al., 2017), which in turn may prevent their partners’ exploration.

Accordingly, we hypothesized that the actor’s attachment insecurity would be negatively associated with his/her own level of exploratory behaviors (indexed by engagement, enjoyment, and distress in this study) during the collaborative activity, and the partner’s attachment insecurity would also negatively predict the individual’s exploratory behaviors. Besides, we further assumed that attachment avoidance plays a more important role than attachment anxiety in predicting exploratory behaviors among older adults.

## Materials and Methods

### Participants

The participants were 49 older couples (98 individuals) recruited from communities in Beijing, China. They were currently in their first marriage and have been married for at least 20 years. None of the participants exhibited cognitive impairment or emotional disorders in screening tests, including the Mini-Mental State Examination (MMSE; Cockrell and Folstein, 1987) and the Chinese version of the 15-item Geriatric Depression Scale (GDS-15; Burke et al., 1991). Husbands’ and wives’ mean ages were 70.49 (*SD* = 5.28) and 66.98 (*SD* = 4.54) years, respectively. The average years of education attained by husbands and wives were 13.27 (*SD* = 2.75) and 11.94 (*SD* = 2.51), respectively, and the mean duration of participants’ marriages was 42.05 (*SD* = 6.00) years.

A power analysis was conducted by APIMPowerR (Ackerman and Kenny, 2016) for APIM analyses with indistinguishable dyads with power (1-β) set at 0.80 and *α* = 0.05. The previous studies examining the relationship between attachment and exploration in younger adults showed the actor effect of attachment with a medium effect size (partial *r* = 0.38; Neustadt et al., 2011). For the partner effect of attachment on exploration, as no previous study can be referred directly, we use the effect size of studies on other behaviors (partial *r* = 0.26; Kane et al., 2007). The analysis indicated the minimum sample size required to detect the predictive actor effect and partner effect of attachment orientations was 24 dyads and 46 dyads, respectively.

### Procedure

This study was carried out in accordance with the recommendations of the ethical standards of the Institutional Review Board of Faculty of Psychology, Beijing Normal University, Beijing, China. The protocol was also approved by IRB of the institution.

Each couple was invited to a laboratory and informed that both members would be participating in a study concerning couple interactions and their behaviors would be videotaped during the study. After obtaining informed consent, husbands and wives were shown to separate rooms to complete the marital attachment questionnaire and report their demographic characteristics (e.g., sex, age, educational level, and marriage duration).

Thereafter, the couples together performed a collaborative activity (i.e., couple sand play). They were arranged to a room equipped with a tray of sand and two cabinets displaying a wide assortments of miniature things (e.g., people, animals, plants, buildings, furniture, vehicles, foods, and stones). Each couple was asked to cooperatively use the miniatures to construct a creative scene on the sand and name the scene together within 5 min. During the activity, the husband and the wife took actions in turns. And in each round, they could take only one action on the sand tray, for example, picking one kind of miniatures (e.g., three trees or one house) from the carbine and placing it on the sand, or moving/removing one that has been placed in the tray. They could take as many turns as they needed to complete their work within the period. The couple was allowed to interact freely during the whole process (e.g., discussing what kind of scene they want to create, which miniature they could use, and how to organize the structure) though “interaction” was not explicitly requested. All participants’ activities were videotaped. Overall, the study took approximately 20 min to complete, and participants received 20 yuan in compensation for their time.

### Measures

#### Marital Attachment

Marital attachment was measured using the Older Adults’ Marital Attachment Scale (OAMAS; Wang et al., 2015). This measurement was the first scale developed to measure marital attachment in older adults. In contrast to the usual two-dimensional measures, three dimensions were used to assess attachment in older people in China (Wang et al., 2015). The scale contains 15 items assessing the following three dimensions: marital attachment anxiety (four items; e.g., “my wife/husband seems to notice me only when I’m angry”), marital attachment avoidance (six items; e.g., “I do not like to stay too close to my wife/husband”), and marital attachment security (five items; e.g., “It is easy for me to be affectionate with my wife/husband”). Responses are provided using a seven-point Likert scale ranging from 1 (strongly disagree) to 7 (strongly agree). The scale has demonstrated satisfactory psychometric properties and validity in predicting marital behavior, such as spousal support and conflict, in previous studies (Wang et al., 2012, 2014). Cronbach’s αs for attachment anxiety, attachment avoidance, and attachment security subscales were 0.69, 0.83, and 0.77, respectively, in the current study.

#### Exploratory Behavior Coding

Each participant’s exploratory behavior was rated by independent raters using a revised coding system designed by Feeney (2007); Feeney and Thrush (2010). There were eight types of behavioral indicators: (1) avoidance of the activity, i.e., frequency and intensity of the behavior that suggested unwillingness to participate in the activity; (2) engagement, i.e., the extent to which individuals appeared absorbed in the activity (e.g., making progress on the structure); (3) laughter, i.e., the degree to which individuals laughed during the activity; (4) enjoyment, i.e., frequency and intensity of exhibition or expression of interest or excitement during the activity; (5) lightheartedness, i.e., the extent to which individuals acted or expressed a carefree, cheerful, and uninhibited attitude regarding the activity; (6) anxiety regarding the activity, i.e., the extent to which individuals expressed or exhibited anxiety or distress (e.g., sad facial expressions or nervous fidgeting) during the activity; (7) seriousness, i.e., the degree to which individuals treated the activity as a weighty or important task (e.g., appearing uptight or stiff during the activity); and (8) negative affect during activity, i.e., nonverbal or verbal negativity regarding the activity or the expression of dissatisfaction with the activity. The frequency and quality of each behavior were rated from 1 (not at all) to 5 (of the highest quality and observed constantly).

To establish reliability, four coders, blinded to the study hypotheses, independently coded 24% of the videotapes (randomly selected). All the videotapes were coded twice by each rater, one time for the coding of the wife’s behavior and the other time for the coding of the husband’s behavior. The intraclass correlation coefficients (ICCs) were then calculated to evaluate the consistency across the raters. All the ICCs were larger than 0.80 which reached the criteria (the ICCs for each coding are listed in Table 1). The remained 76% of the tapes were coded by the four coders independently. For each tape, two raters were assigned to code the wife’s behavior, and the other two raters coded the husband’s behavior. The assignment was random. Any inconsistent coding was discussed till an agreement was reached by the two raters who coded the same participant’s behavior. The means and standard deviations for each coding item are given in Table 1.

**Table 1:** The coding scores and ICCs for husbands and wives.

	Husbands		Wives	
	*M* (*SD*)	ICC	*M* (*SD*)	ICC
Enjoyment	2.01 (0.94)	0.87	2.43 (0.93)	0.89
Laughter	1.32 (0.53)	0.84	1.61 (0.72)	0.88
Lightheartedness	1.50 (0.74)	0.88	1.43 (0.64)	0.82
Anxiety with activity	1.52 (0.60)	0.85	1.70 (0.76)	0.87
Seriousness	2.12 (0.97)	0.82	2.07 (0.87)	0.83
NA toward activity	1.15 (0.36)	0.86	1.29 (0.44)	0.84
Avoidance to activity	1.43 (0.74)	0.93	1.19 (0.42)	0.90
Engagement	3.91 (0.86)	0.81	4.10 (0.89)	0.86

## Results

### Preliminary Analyses

To reduce the number of dependent variables, an exploratory factor analysis with the method of principal component analysis (PCA) was performed to identify the sub-dimensions of the eight types of behavior observed. PCA revealed the presence of three components with eigenvalues exceeding 1, explaining 31.14, 20.69, and 15.72% of the variance, respectively, (i.e., 67.55% of the total variance). To aid interpretation of these three components, oblimin rotation was performed. The simple structure after rotation is given in Table 2 (loadings greater than 0.5 were presented only). According to the component matrix, these three sub-dimensions were labeled as enjoyment (including enjoyment, laughter, and lightheartedness; indexing the extent to which individuals were happy with the activity), distress (including anxiety, seriousness, and negative affect; indexing the extent to which individuals showed anxiety or strain during the activity), and engagement (including engagement and the reversed avoidance; indexing the extent to which individuals were involved in the activity). The composite dimensional scores were used in further analyses.

**Table 2:** The construct of coding items based on EFA.

	Factor 1	Factor 2	Factor 3
Enjoyment	0.81		
Laughter	0.79		
Lightheartedness	0.67		
Anxiety with activity		0.84	
Seriousness		0.72	
NA toward activity		0.71	
Avoidance to activity			−0.78
Engagement			0.84

### Exploratory Behavior and Attachment in Older Adults

The means and standard deviations for the attachment orientations and the three dimensions of exploratory behavior in the collaborative activity for husbands and wives are given in Table 3. For descriptive purposes, a repeated-measures ANOVA with the dimension of exploratory behavior as a within-subject factor was conducted for husbands and wives separately to explore the pattern of behavior during collaborative activity. The dimension of exploratory behavior was significant for both husbands, *F* (2, 96) = 307.41, *p* < 0.001, $\eta_{p}^{2}$ = 0.865, and wives, *F* (2, 96) = 361.30, *p* < 0.001, $\eta_{p}^{2}$ = 0.883. The contrast tests with Helmert coding method showed that, generally speaking, the older adults delighted in the activity, as the level of distress during activity was significantly lower than the mean of the other two positive dimensions of exploratory behavior, namely, engagement and enjoyment [husbands: *F* (1, 48) = 158.23, *p* < 0.001, $\eta_{p}^{2}$ = 0.767; wives: *F* (1, 48) = 157.89, *p* < 0.001, $\eta_{p}^{2}$ = 0.767], and the level of engagement was greater than enjoyment [husbands: *F* (1, 48) = 451.54, *p* < 0.001, $\eta_{p}^{2}$ = 0.904; wives: *F* (1, 48) = 757.23, *p* < 0.001, $\eta_{p}^{2}$ = 0.940]. Similar analyses on scores of attachment orientations were separately conducted for husbands and wives. The results revealed, regardless of gender, the level of attachment security was significantly higher than those of the two insecure dimensions, *F*s (1, 48) ≥ 110.84, *p*s < 0.001, $\eta_{p}^{2}$ ≥ 0.698; and when looking at the two insecure dimensions, attachment avoidance was greater than anxiety, *F*s (1, 48) ≥ 4.22, *p*s ≤ 0.045, $\eta_{p}^{2}$ ≥ 0.081.

**Table 3:** The mean scores and standard deviations of attachment dimensions and exploratory behavior for husbands and wives.

Variable	Dimension	Husbands *M* (*SD*)	Wives *M* (*SD*)
Attachment	Avoidance	3.12 (1.20)	3.20 (1.43)
	Anxiety	2.69 (1.32)	2.43 (1.03)
	Secure	5.84 (0.84)	5.52 (0.92)
Exploration	Engagement	4.24 (0.70)	4.45 (0.51)
	Enjoyment	1.61 (0.59)	1.75 (0.62)
	Distress	1.60 (0.53)	1.69 (0.54)

The separate zero-order correlations between attachment and exploratory behavior for husbands and wives are given in Table 4 and reveal that, consistent with attachment theory, attachment security was negatively associated with the other two insecure dimensions. In addition, the correlation matrix revealed that the level of attachment avoidance was associated with exploratory behavior. Specifically, the husband’s avoidance was negatively correlated with his own enjoyment score and the wife’s avoidance was positively related to her partner’s distress level (see Table 4).

**Table 4:** The correlations among study variables.

S.No.		1	2	3	4	5	6	7	8	9	10	11
1	Secure (H)											
2	Avoidance (H)	−0.54^**^										
3	Anxiety (H)	−0.55^**^	0.36^*^									
4	Engagement (H)	−0.02	−0.21	0.02								
5	Enjoyment (H)	−0.01	0.28^*^	−0.02	−0.12							
6	Distress (H)	−0.26^†^	0.21	0.15	0.01	−0.02						
7	Secure (W)	0.29^*^	−0.25^†^	−0.12	0.05	−0.06	−0.37^**^					
8	Avoidance (W)	−0.28^*^	0.14	0.23	0.24	0.09	0.36^†^	−0.49^**^				
9	Anxiety (W)	−0.13	0.00	0.11	0.14	−0.07	0.25^†^	−0.62^**^	0.48^**^			
10	Engagement (W)	−0.09	0.04	0.06	−0.01	0.33^*^	0.01	−0.07	−0.09	−0.05		
11	Enjoyment (W)	−0.04	−0.07	0.15	0.12	0.30^*^	0.01	0.06	0.14	−0.03	0.07	
12	Distress (W)	0.16	0.00	−0.03	0.04	−0.01	−0.08	−0.04	0.18	−0.03	−0.07	−0.03

† *p* < 0.1;

* *p* < 0.05;

** *p* < 0.01;

H, husband’s score; and W, wife’s score.

### Actor and Partner Effect of Attachment on Exploratory Behavior

The hypotheses were tested using the APIM (Kenny et al., 2006), which is a dyadic data analysis technique to estimate the effects of both partners’ attachment orientation on exploratory behavior by separating “actor” and “partner” effects, *via* mixed models in SPSS. Before the APIM analyses, a series of distinguishability tests were employed. The results revealed there was no empirical evidence that dyad members should be differentiated by their gender [distress: *χ*^2^(7) = 8.42, *p* = 0.297; engagement: *χ*^2^(7) = 6.14, *p* = 0.524; and enjoyment: *χ*^2^(7) = 8.93, *p* = 0.257]. Accordingly, indistinguishable APIM was used here and gender was not included in the models. In the current study, the “actor effect” referred to the influence of participants’ attachment orientation on their own behavior during the collaborative activity, while the “partner effect” referred to the influence of participants’ attachment orientation on their partners’ behavior in the collaborative activity. Participants’ age, length of marriage, and education level were not significantly associated with the exploratory behavior (−0.25 ≤ *r*s ≤ 0.22, *ns*), so they were not considered further.

Three indistinguishable APIM analyses were performed to determine whether actors’ or partners’ attachment orientation was associated with participants’ engagement, enjoyment, and distress in the collaborative activity (see Table 5). For engagement, no attachment effects were significant for avoidance, anxiety, or security on the engagement in collaborative activity (*p*s ≥ 0.079). The mean engagement ratings (husbands: *M* = 4.24; wives: *M* = 4.45) indicated that all participants were absorbed in the activity, and the creation of the sand structures progressed steadily. However, attachment scores were associated with levels of enjoyment of the activity. Specifically, actors’ attachment avoidance was positively associated with actors’ own enjoyment of the activity (*b* = 0.30, *SE* = 0.12, *t* = 2.44, *p* = 0.017). In addition, partners’ attachment avoidance predicted individuals’ distress during the collaborative activity (*b* = 0.26, *SE* = 0.12, *t* = 2.25, *p* = 0.027), indicating that individuals with a highly avoidant partner tended to exhibit greater concern and frustration with their own performance during the activity.

**Table 5:** The actor and partner effects of attachment orientation on exploratory behavior.

	Engagement		Enjoyment		Distress	
	*b*	*SE*	*b*	*SE*	*b*	*SE*
Intercept	0.00	0.10	0.00	0.11	−0.02	0.09
*Avoidance*						
Actor effect	−0.21	0.12	0.30^*^	0.12	0.14	0.12
Partner effect	0.18	0.12	0.003	0.12	0.26^*^	0.12
*Anxiety*						
Actor effect	−0.06	0.12	−0.12	0.12	−0.04	0.12
Partner effect	0.16	0.12	0.10	0.12	0.04	0.12
*Secure*						
Actor effect	−0.20	0.13	0.08	0.13	−0.08	0.13
Partner effect	0.19	0.13	0.08	0.13	0.01	0.13

* *p* < 0.05.

## Discussion

The purpose of this study was to examine the associations between attachment orientations and exploratory behaviors in a collaborative novel activity performed by older couples. Knowledge-oriented activity decreases in retired older adults, but the salience of the motivation to maintain close relationships increases (Lang and Carstensen, 1994; Kooij and Zacher, 2016). Accordingly, managing problems in their daily lives with their spouses could become one of the most important and common means of exploring the external world. The current study revealed that, regardless gender, older adults can be highly engaged in such activity. This is consistent with previous findings showing that older adults can participate in a lot of shared activities with their partner (Fitzpatrick, 2009). In addition, the current findings extended the literature regarding the effects of attachment on exploration from early adulthood to later life and from the workplace to family life. The effects of attachment on older adults’ exploratory behavior are discussed below, followed by descriptions of the strengths and limitations of the study and suggestions for future research.

With regard to the exploratory behavior observed in couples during the collaborative activities, two main conclusions can be drawn to support the role of attachment in exploration. First, the results showed that individuals’ attachment avoidance predicted greater enjoyment of their own during the activity. It may be argued that this result is inconsistent with views that attachment insecurity obstructs exploration (Hazan and Shaver, 1990), for example, high avoidance diminishes goal progress (Jakubiak and Feeney, 2016). However, recent research has found that avoidant individuals were more likely to focus on tasks to distant from relationship-related information or event (Andriopoulos and Kafetsios, 2015; Öner and Gülgöz, 2016). Therefore, they might enjoy exploration (e.g., played the sand, selected the miniature figures, or construct the scene) in the collaborative activities as a means of diminishing the interference brought by relationship partner (e.g., avoided discussion with partner), but their performance and goal progress might be inferior to those of secure individuals (Jakubiak and Feeney, 2016).

Second, individuals whose partners were highly avoidant exhibited greater distress in the collaborative activity, compared to those whose partners were not avoidant. Previous research has demonstrated that the availability of attachment figures can alleviate individuals’ distress (Van Well and Kolk, 2008; Karremans et al., 2011; Zilcha-Mano et al., 2012; Gillath and Karantzas, 2015) and facilitates exploratory behavior (e.g., resistance to a challenging task; Feeney and Thrush, 2010). However, individuals with high levels of attachment avoidance, who are physically and emotionally distant from their spouse, could be less likely to cooperate with their partners (Young et al., 2017) and unavailable when their spouses require assistance and support in collaborative activities, leading to their spouse’s higher levels of anxiety and distress toward activity.

It is noteworthy that avoidance was the attachment dimension that was most strongly predictive of exploratory behavior (of both actors and partners). Previous research has also shown that attachment avoidance exerted a stronger negative effect on relationship quality relative to those exerted by other insecure attachment patterns (Jang et al., 2002). This could occur because of the avoidant attachment figure’s unavailability in the relationship. In a series of examinations of the connection between attachment figures’ reactions and exploration within romantic relationships, Feeney (2004, 2007); Feeney and Thrush (2010) found that avoidant attachment figures were usually unavailable when their partners required assistance and support, which reduced their partners’ persistence on exploration.

It is also interesting to note that attachment anxiety did not exert an effect on exploratory behavior. Consistent with previous findings (Fiori et al., 2009; Jain and Labouvie-Vief, 2010), this study showed older adults were likely to report a relatively low level of attachment anxiety, which could have contributed to the absence of the main effect of attachment anxiety. Another possible explanation could involve an ambiguous attachment model that reflects anxiously attached adults. According to attachment theory, anxious individuals usually have available but inconsistent responsive attachment figures. Consequently, their attachment model is based on both positive and negative attachment experiences. Furthermore, empirical studies have shown that anxious individuals’ behavior toward their partners and activities was ambivalent. For example, they could attack their partners (Marchand, 2004) but sometimes adopt an obliging strategy during marital conflict (Shi, 2003). Therefore, ambivalence in anxious adults could have obscured the effects of attachment on exploratory behavior.

The current study has several strengths. For example, to our knowledge, it was the first observational study in which a series of exploratory behaviors were assessed to examine the extent to which attachment orientation predicts exploration among older adults. With regard to older adults, collaborative activities with their spouses in daily life could become common and typical means of exploration. The findings indicating that attachment was associated with older adults’ exploratory behavior supported the idea that attachment processes characterize individuals “from cradle to grave” (Bowlby, 1973). In addition, the study created a typical challenge (i.e., solving a problem with a partner) in older adults’ daily lives to examine the effects of attachment on their behaviors. Most previous studies examining marital and family issues investigated the effects of attachment in the context of a serious threat, such as conflict (e.g., Overall et al., 2015); however, this becomes increasingly uncommon in later life (Smith and Ng, 2009; Wang et al., 2012). Therefore, the current study demonstrated good ecological validity. Last but not least, the current study demonstrated the different roles of individuals’ own and their partners’ attachment in predicting exploratory behavior in a sample of older couples.

Despite these strengths, the study was subject to some limitations. First, caution should be exercised in generalizing the findings to other situations. The collaborative activity used in the current study was a compulsory task, which could have increased levels of engagement and cooperation in both partners. However, some people might not collaborate in activities with their partners in real life. Second, with the relatively small sample size, the current study only revealed the association between attachment orientation and exploratory behavior among older adults and was not able to examine the underlying mechanism. Previous studies on young adults have shown that, compared to secure adults, insecurely attached adults were less likely to benefit from family happiness and were more likely to be overwhelmed by family concerns which leads to lower work satisfaction (Sumer and Knight, 2001). Furthermore, adults with different attachment orientations tend to show different availability and reactions toward their partners, which in turn influences their partners’ exploration performances (Feeney and Thrush, 2010). However, it is unclear whether there are any mediators in the relationship between attachment and exploration in later life.

Further research should consider the effects of attachment on exploration using a free style of activity task, which could be more suitable to mimic interactive and exploratory behavior in real-life contexts (i.e., the intention to cooperate with one’s partner may be presented and observable). In addition, future research should adopt a larger sample to explore the possible mechanism *via* which attachment orientation shapes individuals’ exploratory behavior, which could improve our understanding of the relationship between the attachment behavior system and other behavior systems from a life span perspective.

In summary, the results of the current study suggested that attachment, especially attachment avoidance, played an important role in exploratory behavior in later life. An individual with higher avoidance tends to show more enjoyment but increase his/her partner’s distress during the collaborative activity.

## Data Availability Statement

The raw data supporting the conclusions of this article will be made available by the authors, without undue reservation.

## Ethics Statement

The studies involving human participants were reviewed and approved by the Institutional Review Board of Faculty of Psychology, Beijing Normal University, Beijing, China. The patients/participants provided their written informed consent to participate in this study.

## Author Contributions

YW, FL, and DW wrote the proposal, designed the study, and completed the final draft. YW was involved in data collecting, coder training, and data analyzing. XC and DW commented on the draft. All authors commented on the final manuscript, which was completed by YW, XC, FL, and DW.

### Conflict of Interest

The authors declare that the research was conducted in the absence of any commercial or financial relationships that could be construed as a potential conflict of interest.
